# A Case of Slight Sprain of the Ankle, Cured in Five Days by Rest in a Fracture Box and Emollient Applications

**Published:** 1839-04-20

**Authors:** 


*A case of slight Sprain of the Ankle, cured in five days by rest in a fracture box and emollient applications.*

Two cases of dislocation of the humerus into the axilla were admitted, both caused by falls upon the top of the shoulder while the men were intoxicated. They were readily reduced by placing a towel over the edge of the acromion process, which was given to an assistant with directions to make counter extension across the body in a direction a little above the opposite shoulder. The acromion being fixed, another towel was placed in the axilla to lift the head of the bone over the edge of the glenoid cavity. With the heel in the axilla and two men to make extension at the wrist the dislocation was readily reduced. The cavicle apparatus was then applied, and the arm kept quiet for a few days, when both were discharged well.

Admitted, a case of severe burn of the arms, head, and upper part of the body, in an old woman of 70 years, from her clothes taking fire. Her arms were poulticed and the face and body dressed with the linament of lime-water and linseed-oil. Brandy punch and opiates were given. Death on the third day.

A case of the necrosis of the alveolar processes of the symphysis of the lower jaw, in a child ten years old, from the action of mercury, was admitted. The piece being loose, was removed by Dr. Randolph, with a pair of forceps, and some lint applied to stop the bleeding. The boy was discharged the same day.

---

Profs. Bush and Peter of Lexington, and Dr. H. H. Smith of this city, are among the passengers in to-day's packet for Liverpool.

